# 

**Published:** 1841-01

**Authors:** 


### CONTENTS OF No. XXI.

OF THE

### British and Foreign Medical Review.

JANUARY, 1841.

---

### PART FIRST.—Analytical and Critical Reviews.

PAGE

ART. I.—1. On the Management of the Poor in Scotland. By WILLIAM PULTENEY ALISON, M.D. F.R.S.E. . . . . . . 1

2. Report on the Sanatory State of the Labouring Classes, as affected chiefly by the Situation and Construction of their Dwellings in and about the Metropolis ib.

3. Papers relating to the Noxious Effects of Fetid Irrigations around the City of Edinburgh . . . . . . . . ib.

4. An Examination of the Statements contained in the Papers relating to the Fetid Irrigations around the City of Edinburgh. By WILLIAM TAIT . . ib.

5. Vital Statistics of Glasgow. By ROBERT COWAN, M.D. . . . ib.

6. Hygiène Publique, ou Mémoires sur les Questions les plus importantes de l'Hygiène, appliquée aux professions et aux travaux d'utilité publique. Par A. J. B. PARENT-DUCHATELET . . . . . . ib.

Public Hygiène, or Memoirs on the most Important Questions of Hygiène, applied to professions and to works of public utility. By A. J. B. PARENT-DUCHATELET.

ART. II.—Mémoires sur l'Empoisonnement. Par M. ORFILA . . . 37

Memoirs on Poisoning. By M. ORFILA.

ART. III.—A Practical Work on the Diseases of the Eye, and their Treatment, Medically, Topically, and by Operation. By FREDERICK TYRREL . 55

ART. IV.—1. On Instinct. By W. P. ALISON, M.D. . . . . 90

2. The Philosophy of Instinct and Reason. By J. STEVENSON BUSHNAN, M.D. . ib.

3. On the Habits and Instincts of Animals. By WILLIAM SWAINSON, F.R.S. . ib.

ART. V.—1. A Narrative of the Treatment experienced by a Gentleman during a state of Mental Derangement; designed to explain the Causes and the Nature of Insanity, and to expose the injudicious conduct pursued towards many unfortunate sufferers under that calamity . . . . 104

2. A Narrative of the Treatment experienced by a Gentleman during a state of Mental Derangement; designed to explain the Causes and the Nature of Insanity, and to expose the injudicious conduct pursued towards many unfortunate sufferers under that calamity. By JOHN PERCEVAL, Esq. . ib.

3. Aphorisms on the Treatment and Management of the Insane; with Considerations on Public and Private Lunatic Asylums, pointing out the errors in the present system. By J. G. MILLINGEN, M.D. . . . ib.

4. Bemerkungen über den Umgang mit Geistkrankheiten, für diejenigen die mit ihnen in Berührung kommen. VON J. F. LEHMANN . . . ib.

Remarks on the conduct to be observed towards Lunatics by those who are in charge of them. By J. F. LEHMANN.

5. Du Danger des Rigueurs Corporelles dans le Traitement de la Folie. Par le Docteur BLANCHE . . . . . . ib.

Of the Danger of Corporal Severities in the Treatment of Insanity. By Dr. BLANCHE.

6. The Fifty-fifth Report of the Visiting Justices of the County Lunatic Asylum at Hanwell; and Second Report of the Resident Physician (Dr. CONOLLY) . ib.

ART. VI.—1. Elements of Chemistry, including the application of the Science in the Arts. By THOMAS GRAHAM, F.R.S. L. & E. . . . 129

2. Chemistry of Organic Bodies: Vegetables. By THOMAS THOMSON, M.D. &c. ib.

3. An Introduction to the Study of Chemical Philosophy; being a preparatory View of the Forces which concur to the production of Chemical Phenomena. By J. FRED. DANIELL, F.R.S. . . . . . . ib.

§

4288

PAGE

4. Supplement to the Introduction to the Atomic Theory; comprehending a Sketch of certain Opinions and Discoveries bearing upon the general principles of Chemical Philosophy which have been brought into notice since the publication of that work. Prefaced by some Remarks on the projected Reforms in Academical Education. By CHARLES DAUBENY, M.D. F.R.S. &c. 129

ART. VII.—Acute Hydrocephalus, or Water in the Head, an Inflammatory Disease, and curable equally and by the same means with other diseases of Inflammation. By DAVID D. DAVIS, M.D. M.R.S.L. . . . 151

ART. VIII.—Cancer. By WALTER HAYLE WALSHE, M.D. (Cyclopædia of Practical Surgery, Parts VI-VII.) . . . . . 158

ART. IX.—Das Schräg-verengte Becken nebst einem Anhange über die wichtigsten Fehler des weiblichen Beckens überhaupt. Von Dr. F. C. NAEGELE 167
The Obliquely-contracted Pelvis, with an Appendix treating of the principal Defects of the Female Pelvis. By Dr. F. C. NAEGELE, &c.

ART. X.—1. A Practical Treatise on the Diseases peculiar to Women; illustrated by Cases derived from Hospital and Private Practice. By SAMUEL ASHWELL, M.D. . . . . . . 174
2. A Practical Treatise on the Function and Diseases of the Unimpregnated Womb. Illustrated by Plates, &c.; with a Chapter on Leucorrhœa, Fluor Albus, or "Weakness." By CHARLES WALLER, M.D. . . ib.

ART. XI.—Statistical Reports on the Health of the Navy, for the years 1830-1-2-3-4-5-6. (South American, West Indian and North American, Mediterranean, and Peninsular Commands.) By Dr. JOHN WILSON, R.N. . . 182

ART. XII.—La Chirurgie de M. Dieffenbach. Par CHARLES PHILLIPS . 194

The Surgery of M. Dieffenbach. By CHARLES PHILLIPS.

ART. XIII.—1. A Treatise on the Diseases of Infants; founded on recent Clinical Observations and Investigations in Pathological Anatomy, made at the Hospice des Enfans-Trouvés; with a Dissertation on the Viability of the Child. By C. M. BILLARD, M.D. &c. With Notes, by Dr. OLLIVIER. Translated from the third French Edition, with an Appendix, by JAMES STEWART, M.D. 198
2. A Practical Treatise on the Management and Diseases of Children. By R. S. EVANSON, M.D. &c., and HENRY MAUNSELL, M.D. &c. . . ib.

ART. XIV.—The Anatomy of the Arteries of the Human Body; with its Applications to Pathology and Operative Surgery; in Lithographic Drawings: with Practical Commentaries. By RICHARD QUAIN. The Delineations by JOSEPH MACLISE, Esq. . . . . . . 210

ART. XV.—Die Krankheiten des Foetus. Von Dr. J. GRAETZER . . 212

The Diseases of the Fœtus. By Dr. J. GRAETZER.

### PART SECOND.—Bibliographical Notices.

ART. I.—The Pathology and Diagnosis of Diseases of the Chest, comprising a Rational Exposition of their Signs; with an Appendix containing various opinions and experiments on the Motions and Sounds of the Heart and on the Bronchi. By CHARLES J. B. WILLIAMS, M.D. F.R.S. . . 217

ART. II.—Remarks on the Surgical Practice of Paris. Illustrated by Cases. Being a Thesis to which a gold medal was assigned by the Senatus Academicus of the Edinburgh University at the Graduation of 1840. By W. O. MARKHAM, M.D. . . . . . . 218

ART. III.—Demonstrations of Anatomy; being a Guide to the Dissection of the Human Body. By GEORGE VINER ELLIS . . . . 220

ART. IV.—Cursory Notes on the Morbid Eye. By ROBERT HULL, M.D. . 221

ART. V.—A Probationary Essay on the Special Pathology of the Accessory Organs of Hearing; submitted by the Authority of the President and his Council to the examination of the Royal College of Surgeons of Edinburgh, when Candidate for admission into their body, in conformity to their Regulations respecting the Admission of Ordinary Fellows. By JAMES MERCER, M.D. . 222

PAGE

ART. VI.—Derangements, primary and reflex, of the Organs of Digestion. By ROBERT DICK, M.D. . . . . . . 223

ART. VII.—The Maternal Management of Children, in Health and Disease. By THOMAS BULL, M.D. . . . . . . 224

ART. VIII.—Elements of Geology for popular Use; containing a Description of the Geological Formations and Mineral Resources of the United States. By CHARLES A. LEE, M.D. . . . . . . ib.

ART IX.—A Treatise on the Structure, Functions, and Diseases of the Foot and Leg of the Horse; comprising the Comparative Anatomy of these parts in other Animals, &c. By W. C. SPOONER, M.R.V.C. . . . ib

ART. X.—The Retrospect of Practical Medicine and Surgery for the year 1840; being an analysis of the British and Foreign Medical Journals and Transactions, or a Selection of the latest Discoveries and most Practical Observations in the practice of Medicine, Surgery, and the collateral Sciences for the past year; made chiefly with reference to the Treatment of Disease. By W. BRAITHWAITE, M.R.C.S. . . . . . . ib.

---

### PART THIRD.—Selections from the British and Foreign Journals.

### I. THE FOREIGN JOURNALS.

#### ANATOMY AND PHYSIOLOGY.

Dr. MANDL's Memoir on the Relations which exist between Blood, Pus, Mucus, and Epidermis . . . . . . 225

Dr. LENS on Menstruation in a Child . . . . . . ib.

Professor CATTANEI DI MOMO on the natural existence of Copper and of Lead in the Human Body . . . . 226

Dr. TADDEI DE GRAVINA's Researches on the Specific Odour of the Blood . ib.

Dr. OESTERLEN on the Extraordinary Enlargement of the Kidneys in the Fœtus, from the Development of Hydatids in their Substance . . . 227

Professor NASSE on the Microscopic Constituents of Milk . . . 228

Mr. BRESCHET's Experimental Researches relating to the Mode of Transmission of Rabies . . . . . . . 229

Professor MONDINI on Bronchocele in a Fœtus of about Eight Months . . ib.

#### PATHOLOGY, PRACTICAL MEDICINE, AND THERAPEUTICS.

M. RENAULT on the Development of Acute Glanders by the Introduction of Pus into the Veins of a Horse . . . . . 230

Dr. BUDGE on Irritation of a Branch of the Trigeminus Nerve at its central extremity . . . . . . . ib.

Dr. RITTER on the Effects of Over-eating . . . . 232

Dr. LÖWENHARDT on the Medical Action of the Bezoar of the Lacrymal Fossa of the Deer (Deer's tears) in Nervous Diseases . . . 233

Dr. CHAERETES on the Cure of a Severe Gouty Pain by the Electric Ray . ib.

SEMENTINI on the Uncertainty of the Signs of Peritonitis . . . 234

TERRONE's Case of Convulsions of a peculiar kind . . . . ib.

Dr. RITTER on Apoplexy, with Hemiplegia, in consequence of Fright . . 235

Dr. FIORE on the Therapeutic Efficacy of Ammoniuret of Copper in Chorea . ib.

M. RASPAIL on Sedative Lotions in Headaches, Congestions, and Cerebral Fevers ib.

Dr. CHANSAREL's Case of Posthumous Variola . . . . 236

M. VOISIN's Mode of Resolving Engorgements of the Spleen . . . ib.

Professor MORELLI on Graduated Compression of the Abdomen by Bandages . 237

Dr. TRIBERTI on Hydrophobia, and the relation between the Retina and the Respiratory Nerves in that Disease . . . . . ib.

GRANDONI on a stony Concretion in a Human Nose . . . 238

Dr. GALLARDI on Angina Pectoris . . . . . 238

Dr. POPKEN on Tubercles of the Larynx and Fauces . . . 239

Dr. PHILIPP on Dropsy after Scarlatina, unaccompanied with Albuminous Urine . 240

PAGE
Dr. Pogliaghi's Case of Idiopathic Lymphadenitis . . . . 241
MM. Andral and Gavarret on the State of the Blood in different Diseases . 242
M. Leroy-D'Etiolles on Acute Ovaritis induced by Uterine Injections . 246

SURGERY.

Dr. de la Harpe on Itch and its Treatment . . . . ib.
M. Chomel on the Use of Mercurial Plaster in Smallpox . . . ib.
M. Gimelle on the Employment of large Doses of Tartarized Antimony in the Treatment of Articular Dropsies . . . . . 247
Dr. Meding's Case of immense Cæcal Fistula in the Lumbar Region . . 248
M. Guerin on Subcutaneous Section of Forty-two Muscles, Tendons, or Ligaments practised the same day, on the same person, to remedy an articular deformity 250
M. Bouvier on a New Species of Torticollis . . . . ib.
Dr. Schütte on a Wound of the Urinary Bladder . . . . 251
M. Trousseau on the Treatment of Fissures of the Anus by Rhatany . . ib.
Dr. Ruppius on the Use of Boiling Water in the Treatment of Callous Fistulæ . 252
M. Velpeau's Method of Operating for the radical Cure of Hernia . . ib.
Dr. Schilling on Solution and Absorption of Provisional Callus in Typhus Fever 253

MIDWIFERY.

Dr. Schultzen's Case of Fracture of the Skull during difficult Parturition . ib.

CHEMISTRY, PHARMACY, &c.

M. Faucher's Preparation of Mercurial Ointment in Six Hours . . 254
Professor Nasse on Cholestearine in Morbid Fluids . . . ib.

---

### II. THE AMERICAN AND COLONIAL JOURNALS.

Dr. Dunglison on Coagulation of the Blood fifteen hours after Death . . 255

Dr. Norris's Statistical Account of the Cases of Amputation performed at the Pennsylvania Hospital from January 1, 1838, to January 1, 1840 . 256
Dr. Hayward's Statistics of the Amputations of Large Limbs that have been performed at the Massachusett's General Hospital; with Remarks . . 257
Bell on the Effects of Temperance Societies, in diminishing Sickness and Mortality among the Troops in India . . . . . 259

---

### III. THE BRITISH JOURNALS.

Dr. Oliver's Case of Transfusion in Uterine Hemorrhage . . . 260
Smith's Observations on the Diagnosis and Pathology of Fractures of the Neck of the Femur . . . . . . . 262
Dr. Graves on Nervous or Spasmodic Asthma . . . . 264
Clay on the Use of Tincture of the Muriate of Iron in Diabetes Mellitus . 265

---

### Part Fourth.—Medical Intelligence.

London Mortality Tables for the Quarter ending September 26 . . 267
Dr. Carswell . . . . . . . 269
The Claims of Italy to the Invention of Lithotrity vindicated . . . 270
The Queen Adelaide Fund of the Hanwell Lunatic Asylum . . . 271
Earl Spencer on the Gestation of Cows . . . . . 273
Books received for Review . . . . . 275
APPENDIX—Thackeray Prize Essay.

